# Food offerings on board and dietary intake of European and Kiribati seafarers - cross-sectional data from the seafarer nutrition study -

**DOI:** 10.1186/s12995-018-0190-0

**Published:** 2018-02-27

**Authors:** Birgit-Christiane Zyriax, Robert von Katzler, Bettina Jagemann, Joachim Westenhoefer, Hans-Joachim Jensen, Volker Harth, Marcus Oldenburg

**Affiliations:** 1 Preventive Medicine and Nutrition, Institute for Health Services Research in Dermatology and Nursing (IVDP), University Medical Center Hamburg-Eppendorf, Martinistr. 52 - Bldg. O56 – D-20246, Hamburg, Germany; 2 Department of Maritime Medicine, Institute for Occupational and Maritime Medicine (ZfAM) Hamburg, University Medical Center Hamburg-Eppendorf, Hamburg, Germany; 3 I. Medical Clinic and Polyclinic; University Medical Center Hamburg-Eppendorf, Hamburg, Germany; 4 Competence Center Health, Faculty of Life Sciences, Hamburg University of Applied Sciences, Hamburg, Germany

**Keywords:** Seafarer, Kiribati, Diet, Food offerings, Food choice, Anthropometry

## Abstract

**Background:**

Overweight and cardiovascular risk factors are a common phenomenon in seafarers. According to internal observation particularly crew members from the Pacific Island State of Kiribati are exposed to a high risk. However, in mixed crews, cultural background plays an important role, influencing food choice, and the actual risk.

**Methods:**

The Seafarer Nutrition Study (SeaNut study) compared dietary factors in 48 Kiribati and 33 European male seafarers recruited from four merchant ships with a high level of Kiribati manning within a German shipping company. Analysis encompassed the assessment of dietary quality on board, satisfaction with prepared dishes, and individual food intake obtained from 24-h recalls in comparison with nutritional recommendations.

**Results:**

The overall supply of meat, fat and eggs was more than double, whereas the proportions of fruits, vegetables, dairy products and cereals were much lower than recommended. Based on the reported food choices, both groups, but notably Kiribati seafarers, did not reach reference values as to macronutrient, micronutrient and fiber intake. In addition, satisfaction with the meals served, food preferences and knowledge about a healthy diet varied markedly between Kiribati and Europeans.

**Conclusions:**

The present analysis of the SeaNut study revealed the necessity of future health intervention programs, including the quality of the food supply as well as information about a healthy diet and adequate food selection. In mixed crews, culture-specific differences should be considered, in order to facilitate the long-term success of interventions.

**Trial registration:**

German Clinical Trials Registry DRKS00010819 retrospectively. Registered 18 July 2016 (www.germanctr.de).

## Background

Previous research indicates that seafarers on merchant ships are exposed to a high risk of overweight and cardiovascular disease due to specific work conditions [1]. Obesity not only increases the risk of cardiovascular events but also affects physical performance in daily duties and safety of operations and may become a problem in cases of acute complications without adequate access to professional medical service [2, 3]. Restricted food choice, limited leisure time possibilities, and smoking in addition to psychosocial stress and homesickness, characterize the specific workplace conditions, and the risk appears to increase with increasing duration of employment at sea [4–6]. However, according to an internal report of a German shipping company particularly crew members from the Pacific Island State of Kiribati are affected by a substantial weight gain during their stay on board. Currently more than 60,000 seafarers are employed on German vessels with a share of at least 2% from this very small Pacific Island. In Germany, there are some shipping companies with a high proportion of Kiribati seafarers as these employees are known to be very experienced in seafaring and medical fit for sea service. The social-economic status of Kiribati seamen is generally low, corresponding with their often low-rank position on board as non-officers.

Predominantly limited opportunities to select a healthy, balanced diet for several months on board may contribute to the high prevalence of overweight and associated cardiovascular risk factors among seafarers [1, 2]. Due to the stressful working conditions, eating is an important daily pleasure. A survey of two Danish shipping companies in a homogeneous collective reported a higher frequency of overeating as well as a higher intake of cakes, sweets and sugared beverages on board compared with the home setting [7]. In addition, previous research indicates that the traditional diet on board seems to be meat-oriented, while the consumption of fresh fruits and vegetables is rather low [8, 9].

Overall, research providing a detailed assessment of food offerings on board, as well as satisfaction with prepared meals and individual food choice in multi-ethnic crews is limited. This prompted us to investigate the Seafarer Nutrition Study (SeaNut) to analyze food quantity and quality on merchant vessels in relation to current recommendations and to compare individual nutrition intake, food preferences and satisfaction of European and Kiribati seafarers in order to identify unhealthy dietary habits.

## Methods

### Design and recruitment

The SeaNut study is a cross-sectional study comparing dietary and lifestyle factors, anthropometric parameters in seafarers of European origin with those from Kiribati, a Pacific island group. Between April and August 2014, over a course of more than 100 days a total of 81 male seafarers, 33 from European countries (i.a. 18 from Germany, 9 from Poland) and 48 Kiribati, were recruited from four merchant ships of a German shipping company during the sea voyage. Since the shipping company tries to assign groups of seafarers of the same origin on their ships, 4 vessels with a particular high number of Kiribati were identified for examination. The study protocol was approved by the Ethics committee of Medical Association Hamburg and conducted according to the principles of the Declaration of Helsinki. Written informed consent was obtained from all participants.

### Data collection

All interviews and physical examinations were taken on board of the ships and conducted in English, the official language on board and performed by the same previously trained physician.

#### Assessment of demographic and clinical data

Information about demographic characteristics, occupation, medication use, and family history of cardiovascular disease was obtained using questionnaires. Body height and weight were measured during the ship’s stay in port. Body mass index (BMI) was calculated as BMI = (weight, kg)/(height, m)^2^. Overweight was defined as a BMI ≥ 25 kg/m^2^, while a BMI ≥ 30 kg/m^2^ was considered as obesity. To assess the weight development of the Kiribati crew members up to the current shipboard examination, information about weight and height from their prior medical fitness tests ashore were obtained. Waist circumference (WC) was measured halfway between the lower rib margin and the iliac crest. A waist circumference > 94 cm was defined as central obesity, however cut-off values referred to Europeans as no reference-values were available for Kiribati in the literature.

#### Assessment of food offerings on board and individual satisfaction

**Fig. 1:**
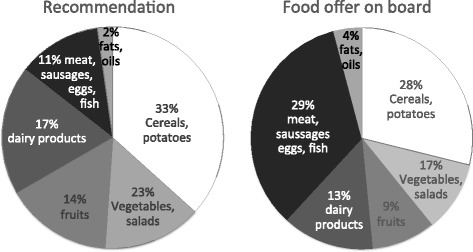
Proportional distribution of food groups on board in comparison with nutritional recommendations. The analysis comprised the proportional intake of six relevant food groups representing a healthy balanced diet according to nutritional recommendations [10]

To assess the quality and quantity of the diet on board, the preparation and the composition of the three main courses per day were analyzed on every ship on seven consecutive days, taken into account recipes and the proportional distribution of relevant food groups. Meal components were weighted and a ‘standard plate’ representing the average offering on board was calculated and photographed. Since special nutritional guidelines for seafarers and particularly reference values for Kiribati are lacking the analysis mainly comprised the proportional intake of six relevant food groups representing a healthy balanced diet according to nutritional recommendations: Cereals and potatoes (33%), vegetables and salads (23%), dairy products (17%), fruits (14%), meat, processed meat, eggs and fish (11%), and finally, fat and oils (2%) (Fig. 1) [10]. Apart from an additional pan with freely available rice the food provision for officers and non-officers did not differ aboard the four examined vessels.

Regarding the daily food offerings on board, during a structured interview all seafarers were asked about their satisfaction (single choice question: “Are you satisfied with the supply of food you have on board?”), individual wishes and suggestions (multiple choice question: “Imagine you could make changes related to the food on board. Please check from the following list the items which you would change.”), and in addition their knowledge about a healthy diet (single choice question: “Did you feel well-informed about- healthy food?” Yes-No). These questions were analyzed on a group basis, comparing European vs. Kiribati seafarers [11].

#### Assessment of individual food intake

Individual nutrition intake was obtained by the 24-h dietary recall method, an instrument, that is widely used in surveys and cross-sectional and cohort studies. As an open instrument the tool is suited for the assessment of dietary intake independent of cultural background [12]. This retrospective method consists of recalling and quantifying the intake of all foods and beverages consumed in the 24-h period prior to the interview and is therefore not influenced by shift work. The data were collected via face-to-face interview. The above described “standard-plate” was used as a supporting instrument to improve the assessment of dietary intake by considering portion size. Nutrition assessment followed a standardized protocol [13]. On the average, three non-consecutive 24-h recalls per person were applied (range 2–5). The results were compared with the recommendations of the German, Swiss, and Austrian Nutrition Societies [14], because information about nutrition requirements of the Kiribati population are lacking. Food records from 39 Kiribati and 24 European seafarers were available. In order to achieve a high data quality, the assessed food intake was only used from seafarers with at least two 24-h recalls available. Unfortunately several participants gave only one 24-h recall and were consequently not included. For the evaluation of the nutrition data, the OptiDiet Basic Software version 5.1 was used, which includes 15,000 foods (food items) based on the German food composition database BLS version 3.01 [15].

### Statistical analysis

Baseline characteristics of the two groups (Kiribati and European) are reported as median (min-max) for continuous data or percentages for categorical data and compared using Mann–Whitney U-tests or likelihood ratio chi-square tests, as appropriate. In cases of a smaller number of incidents, Fisher’s exact-test was used. *P*-values below 0.05 were considered to indicate statistically significant results. Statistical analyses were performed using SPSS version 20 (IBM Corporation, New York, NY).

## Results

### Baseline characteristics

Median age did not differ between Kiribati and European crew members (Table 1). Most of the seafarers were married. However, more Kiribati than Europeans reported having one or more child. Occupational position differed significantly. None of the Kiribati but two-thirds of the European seafarers were employed as officers (11 nautical officers, 7 technical officers, 15 engine-room ratings, 48 deck ratings). Among Kiribati, 58% were assigned as deck ratings, 27% as engine-room ratings and 15% as galley personnel. In view of the rather small sample size of the study, the occupational status was not included in further analysis. Compared with Europeans, median BMI and waist circumference were significantly higher in the Kiribati (Table 1). Kiribati were more often characterized by a higher prevalence of smoking. However, the results failed to show significance (Table 1). No differences of demographic or nutrition-related data were observed between the ships.

**Table 1:** Baseline characteristics of the population

Variables	Europeans *n* = 33	Kiribati *n* = 48	Significance *P*-value
Age (years)	33 (20–60)	38 (23–64)	ns ^b^
Family status			
Married (%)	67	77	ns ^a^
≥ 1 child (%)	46	81	0.001 ^a^
Occupational status			
Officer (%)	67	0	< 0.001 ^c^
Non-officer (%)	33	100	
Body mass index (kg/m^2^)	25.4 (18.3–35.0)	30.1 (21.1–40.5)	< 0.001 ^b^
Waist circumference (cm)	93.0 (75–115)	97 (74–125)	0.045 ^b^
Current smokers (%)	47	56	ns ^a^

Values are given as percentage, or median (min-max)

^a^P-value of chi-square test

^b^P-value of Mann–Whitney U-test

^c^P-value of Fisher’s exact-test

### Food offerings on board and individual satisfaction

To assess the quality of the diet offered on board, the composition of the “standard-plate” was compared with nutritional recommendations [10]. The analysis clearly showed that the amounts of meat, processed meat and eggs were approximately triple, while the amount of fats and oils was double the recommendation (Fig. 1). Only once a week fish was offered on board. In contrast, the proportions of offered fruits, vegetables and salads as well as those of dairy products, cereals, and potatoes were below the recommendations (Fig. 1).

**Fig. 2:**
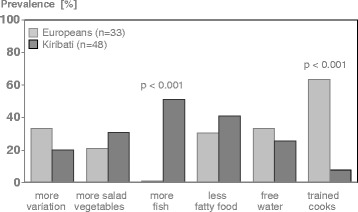
Attitudes towards food offer in Kiribati and European seafarers. Values are given in percentage. *P*-values of chi-square test

Information about individual satisfaction with the food offers on board and food preferences varied markedly depending on cultural background (Fig. 2). While half of Kiribati desired more fish, none of the European seafarers did. More Europeans reported that they would appreciate a better trained cook. However, statements common to both groups focused on a greater variety of food, the preparation of more vegetables and salads, less fatty food and the availability of free mineral water.

The majority of the crew reported that job satisfaction depends on the quality of food offered (Kiribati: 61%; Europeans: 55%). However, only 24% of Kiribati and 30% of Europeans rated the taste of the food as good. Interestingly, 67% of Kiribati and 72% of European seafarers would appreciate consuming a healthier diet on board, but only 54% of Kiribati and 85% of the Europeans felt properly informed about balanced nutrition (*p* = 0.005). Fewer Kiribati agreed with the statement that the preparation of dishes considered culture-specific preferences (data not shown). Both groups reported receiving sufficient food on board (Kiribati: 94%; Europeans: 88%), yet 42% of Europeans and 21% of Kiribati stored food in their cabins (*p* = 0.042; mainly confectionery, fruits and nuts). Notably more Kiribati than Europeans were affected by stomach aches (36% vs 9%; *p* = 0.008) and diarrhea (15% vs 3%; ns) after meals.

### Intake of energy and macronutrients

Daily food intake depends on the offerings on board. However, personal decisions about the amount of food consumed and individual food preferences are possible. Based on the evaluation of the 24-h recalls a higher intake of energy, fat protein and carbohydrates was calculated for Kiribati seafarers, whereas less alcohol consumption was reported (Table 2). With regard to relative energy consumption only the percentage of protein intake differed between Kiribati and European crew members (20% vs 17%). On the average total carbohydrate and fiber intake was lower than recommended in both groups: 75% of Europeans and 87% of Kiribati reported a carbohydrate intake below 50% of total energy consumption. Furthermore, 92% of European and 100% of Kiribati seafarers did not reach the reference value of 30 g fiber per day. In contrast, mean sugar intake (sucrose) was higher than recommended, regardless of cultural background. In addition, Kiribati as well as European seafarers consumed high amounts of fat, particularly saturated fat and cholesterol (Table 2): 67% of Europeans and 92% of Kiribati reported a fat intake above 30% of total energy consumption, while cholesterol intake in 88% of European and all Kiribati seafarers was above the reference value of 300 mg per day. Saturated fat intake in Kiribati as well as in Europeans contributed to 15% of total calorie intake, which is more than double the recommended < 7% of energy intake according to the guidelines of the American Heart Association [16]. In contrast, the intake of polyunsaturated fat, particularly fatty acids of omega-3 origin (e.g., fish, nuts, rapeseed oil) was low, which was also reflected by an unfavorable omega-6 to omega-3 quotient above the recommended ratio of 1.5 (data not shown).

**Table 2:** Self-reported energy and macronutrient intake of European and Kiribati seafarers

Intake per day	Europeans *n* = 24	Kiribati *n* = 39	Significance *P*-value
Energy (kcal)	3,094 (2,008–3,920)	3,315 (2,541–4,253)	0.017
Protein (g)	123.3 (61–180)	161.2 (116–205)	0.001
Carbohydrates (g)	311 (222–432)	357 (193–512)	0.006
Saccharose (g)	59.8 (23.8–140.6)	73.7 (6.1–215.2)	ns
Fiber (g)	21.5 (11.2–38.2)	17.1 (10.8–28.4)	< 0.001
Fat (g)	117.1 (68.7–207.8)	135.9 (75.0–208.1)	0.015
Saturated fat (g)	54.6 (26.8–91.3)	57.3 (22.5–97.4)	0.089
n-6 fatty acids (g)	13.6 (5.2–31.0)	18.6 (10.5–30.9)	0.002
n-3 fatty acids (g)	2.3 (1.3–5.3)	2.4 (1.2–5.0)	ns
Cholesterol (mg)	507 (206–1185)	871 (396–1533)	< 0.001
Alcohol (g)	13.1 (0.1–52.3)	0.5 (0.01–700)	< 0.001
Water (l)	3.0 (1.5–4.9)	2.5 (1.5–4.1)	0.008

Values are given as median (min-max). P-value of Mann–Whitney U-test

### Intake of vitamins and minerals

**Fig. 3:**
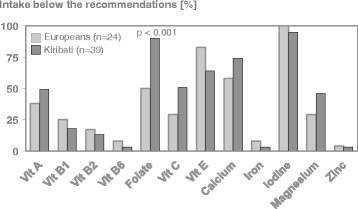
Percentage of European and Kiribati seafarers with micronutrient intakes below the recommendations. Information is based on self-reported dietary intake and compared with nutritional recommendations [13]. P-values of chi-square test

Evaluation of micronutrient intake showed that mean daily intake of folic acid was significantly lower in Kiribati than in Europeans (0.24 (0.13–0.38) mg vs 0.30 (0.14–0.41) mg, whereas mean ingestion of iodine did not differ (Kiribati: 0.12 (0.05–0.20) mg; European: 0.10 (0.05–0.16) mg; ns). However, mean dietary intake of both micronutrients was obviously below the recommendation in both groups (folic acid: 0.30 mg/day; iodine: 0.2 mg/day) [13]. In addition, except for vitamin B12, a considerable portion of seafarers did not reach the recommended threshold (Fig. 3). None of the crew members fulfilled the recommendation as to vitamin D intake. Interestingly, despite exposure to sunlight (UVB), 54% of Kiribati and 76% of Europeans were characterized by vitamin D plasma levels below 20 ng/dl (data not shown). Regarding salt consumption, daily sodium intake of all seafarers exceeded the recommendation of < 2.3 g corresponding to < 6 g salt per day [14, 16]. Besides the intake of micronutrients from food, European seafarers but none of the Kiribati reported using supplements containing vitamins (27%) or minerals (24%). However, evaluation of the obtained data is difficult as various products were reported.

## Discussion

To our knowledge this is the first comprehensive investigation assessing food quality, satisfaction with on-board food availability and individual food choice in European and Kiribati seafarers. Taking into account the six relevant food groups that characterize a healthy diet, one principal finding of the SeaNut study was that the overall food supply on board did not meet nutritional recommendations. However, although both ethnic groups received the same food offerings, individual food choice revealed that the observed mismatch was more pronounced in Kiribati concerning to folate and by trend to vitamin C, calcium and magnesium (Fig. 3). In addition, satisfaction with prepared dishes, individual food preferences, and knowledge about a healthy diet varied markedly between Kiribati and Europeans. Therefore, in mixed crews effective health programs targeting food offerings and individual food choice on board should consider cultural background, which may facilitate or hinder the implementation and long-term success of interventions.

### Food supply and food choice

According to the present analysis, the diet on board was more meat-, and fat-oriented, while fewer fruits, vegetables, dairy products, cereals and potatoes were offered. These findings are largely supported by previous research [7, 9]. According to a Polish study, the energy intake of crew members substantially exceeded the recommended daily level [8]. Based on Danish research, obesity represents a major public health issue among seafarers, and there is a call for corporate action to address its increasing rates [2, 17]. Interestingly, in the SeaNut study, the preferential selection of energy-dense food concomitant with a strikingly high intake of calories, saturated fat, cholesterol, sugar, salt, and less fiber was common, but predominantly observed in the Kiribati. In fact, overall, Kiribati seafarers were more satisfied with the food supply and reported consuming more food during their stay on board than at home. Historically, Pacific Islanders were not overweight and consumed more fish. However, dietary patterns in the Federal States of Mirconesia have shifted. Processed food rich in saturated fat and refined carbohydrates imported mostly from the U.S. contributed to the global rise in obesity [18–20].

According to an internal observation from a previous study the total energy expenditure per day depends on the occupational position (2.880 kcal nautical officers, 3.563 kcal ratings deck and 3.389 kcal engine room personnel) (unpublished data). These data indicate strenuous physical tasks especially for ratings as mainly represented by Kiribati in this study. In spite of this physical effort, Kiribati were characterized by substantial weight gain on board and a much higher BMI and waist circumference in comparison with Europeans. In addition, dietary habits have a major impact on coronary heart disease independent of, and additive to, that of conventional risk factors, which underscores the importance of nutritional interventions on board [21].

### Job satisfaction depends on the quality of the food supply

Of course, food is an important pleasure of the day, and overeating has been described previously as a common phenomenon in seafarers due to specific workplace conditions [7, 22]. Stressful conditions such as shift work, long working hours, less sleep and irregular meal times influence appetite, emotional eating, and food choice. Consequently, a higher amount of energy-dense, fat- and sugar-rich food is preferred [23]. In addition, stress exaggerates diet-induced obesity through neuropeptide Y, leading to the accumulation of abdominal fat [24]. In fact, compared with Europeans, Kiribati were more often identified as having a higher stress level, but the difference was not significant (data not shown). Another relevant determinant of obesity is the socioeconomic condition, e.g., the fact that Kiribati hold lower-ranked positions [25]. However, according to a previous report, a higher frequency of overeating on board did neither depend on professional status nor workplace [22]. Hence, in terms of obesity, cultural background and genetic aspects, which cannot be excluded, appear to play an important role [25, 26]. Recently, a cross-sectional study of another Pacific island society indicated a nutritional transition toward more imported and processed foods and a more sedentary lifestyle associated with a higher prevalence of the metabolic syndrome [27]. Still, more information about eating behaviors of non-Europeans, particularly Kiribati is required.

### A more balanced diet on board is favored

Interestingly, despite their cultural differences, most Kiribati and European seafarers reported that they would appreciate a more balanced diet on board. In line with this statement, both groups asked for the preparation of more vegetables and salads, fewer fatty foods, the availability of free mineral water, and actions to be taken to improve food quality on board. As a consequence of the limited supply of fresh fruits, vegetables, legumes, potatoes, and cereals a consistent number of crew members fail to meet the recommended daily intake of micronutrients such as folate and vitamin C, as well as magnesium. Calcium deficiency reflects the rather low consumption of dairy products by the crew. Certainly, with regard to the high prevalence rate of lactose intolerance in Polynesian populations, calcium sources such as vegetables (e.g., broccoli, cabbage) and calcium-rich mineral water should be considered [28]. Carbohydrate intolerance may also explain in part the higher prevalence of gastrointestinal disorders in Kiribati seafarers compared with Europeans. Nevertheless, it might be necessary to provide improved logistics (e.g. larger fresh food supply aboard ships, more frequent deliveries, larger refrigeration rooms) to accommodate the need for more fresh and healthy food.

### Challenges in promoting a healthy diet on board

Despite favoring a more balanced diet, Kiribati felt less informed about the criteria for good nutrition and were not able to assess whether the prepared meals included a variety of foods. Simplified information provided by a health consultant and adapted to cultural preferences seems to be useful but not at all sufficient. Implementing and maintaining a healthy diet and food choice on board is a huge challenge. Knowledge transfer along with individualized tips, strategies to overcome traditional dietary habits, and improved skills of the cooks, as well as logistical efforts to offer fresh food and free calcium-rich mineral water should be considered and addressed at the management level [7]. In the SeaNut study particularly Europeans suggest better training for cooks. Encouragement and continuous training of seafarers with food responsibilities will contribute better quality and taste to prepared meals, a frequently mentioned desire in both groups. In a pilot project from Finland special attention and information regarding healthier eating habits and less alcohol consumption was provided to seafarers at risk [29]. One year after the intervention alcohol consumption was reduced and an improvement in seafarers’ perception of the meals prepared as being healthier was documented. Another study from Denmark clearly showed that, after a training was provided to cooks, significant changes, such as sugar reduction and more frequent use of vegetables were observed however, the results may not apply to multi-ethnic groups [7].

### Interventions should take cultural preferences into consideration

Results from the SeaNut study indicate that cultural background markedly influences satisfaction with the food supply and individual food choice. Particularly Kiribati claimed that the cook did not consider culture-specific preferences when preparing meals. Despite high satisfaction with meat dishes, Kiribati predominately suggest more frequent preparation of fish dishes, reflecting the traditional diet in their home country. Instead of an often uncontrolled use of nutritional supplements, replacing meat by more fish on board will markedly reduce the intake of saturated fat and cholesterol and contribute to an improved and sufficient supply of iodine, vitamin D, and omega-3 fatty acids. In contrast, seafarers of European origin predominately cited the restricted food choice as one explanation for why they stored food twice as often in their cabins. Of course, in light of a sustainable intervention continuous training of seafarers responsible for food preparation should be considered. A shift towards a lighter, more plant-based nutrient-dense diet comprising higher amounts of fresh fruits and vegetables, fish instead of meat, less fat, salt and sugar will reduce the prevalence of overweight and obesity. In addition, a healthy, well-balanced diet will counteract micronutrient deficiency. The use of iodized table salt will contribute to an improved supply of iodine. Overall, the findings of the SeaNut study underline the need for continuous interventions taking into account multi-ethnic differences. The very rich food offerings on board seems particularly tempting for the Kiribati promoting overconsumption and weight gain.

### Limitations

The present analysis of the SeaNut study has several limitations that need to be addressed. First, the sample size is rather small. In consequence differences between the two groups have to be large to reach significance. Second, the nutrient assessment was obtained from 24-h recalls and thus depended on self-reported data. Therefore, underreporting might have occurred, particularly in overweight and obese seafarers. However, if anything this is more likely to lead to an underestimation of the differences in intake. Thirdly, interpretation and comparison of anthropometry and clinical characteristics between the two groups are limited, because reference values for the Kiribati population are lacking. Despite these limitations, our data provide relevant hypotheses about food offerings on board, satisfaction with the food supply, and individual nutrition intake in light of cultural-specific differences that require further confirmation in a larger cohort.

## Conclusion

The analysis of the SeaNut study clearly showed a mismatch between food supply, food choice and current recommendations in multi-ethnic ship crews. However, differences were more pronounced in Kiribati compared to European seafarers. In summary, a higher quality of food supply, training for the cook, improved logistics to offer more fresh food, increased awareness about the necessity of dietary improvements, and nutritional counseling delivered to all crew members with respect to cultural preferences and beliefs are highly justified. In the light of the increasing global labor migration the results of the SeaNut study may also provide specific information to enhance future interventions at multi-ethnic workings places.

## Abbreviations

BMI: Body mass index
SeaNut study: Seafarer nutrition study
WC: Waist circumference

## References

[1] Oldenburg M, Jensen HJ, Latza U, Baur X (2008). Coronary risks among seafarers aboard German-flagged ships. Int Arch Occup Environ Health.

[2] Hansen HL, Hjarnoe L, Jepsen JR (2011). Obesity continues to be a major health risk for Danish seafarers and fishermen. Int Marit Health..

[3] Nas S, Fiskin R (2014). A research on obesity among Turkish seafarers. Int Marit Health..

[4] Oldenburg M, Jensen HJ, Latza U, Baur X (2009). Seafaring stressors aboard merchant and passenger ships. Int J Public Health.

[5] Chen WQ, Wong TW, Yu TS (2009). Influence of occupational stress on mental health among Chinese off-shore oil workers. Scand J Public Health.

[6] Fort E, Massardier-Pilonchery A, Bergeret A (2009). Alcohol and nicotine dependence in French seafarers. Int Marit Health..

[7] Hjarnoe L, Leppin A (2014). What does it take to get a healthy diet at sea? A maritime study of the challenges of promoting a healthy lifestyle at the workplace at sea. Int Marit Health..

[8] Babicz-Zielinska E, Zabrocki R (1998). Assessment of nutrition of seamen and fishermen. Rocz Panstw Zakl Hig.

[9] Lawrie T, Matheson C, Ritchie L, Murphy E, Bond C (2004). The health and lifestyle of Scottish fishermen: a need for health promotion. Health Educ Res.

[10] German Society for Nutrition (2017), http://www.dge-ernaehrungskreis.de/start. Accessed 25 June 2017.

[11] Westenhoefer J, von Katzler R, Jensen HJ, Zyriax BC, Jagemann B, Harth V, Oldenburg M (2018). Cultural differences in food and shape related attitudes and eating behavior are associated with differences of body mass index in the same food environment: crosssectional results from the seafarer nutrition study of Kiribati and European seafarers on merchant ships. BMC Obesity.

[12] Ngo J, Engelen A, Molag M, Roesle J, García-Segovia P, Serra-Majem L (2009). A review of the use of information and communication technologies for dietary assessment. Br J Nutr.

[13] Gibson RS (2005). Principles of nutritional assessment: Oxford university press.

[14] D-A-CH Referenzwerte für die Nährstoffzufuhr (2015). Deutsche Gesellschaft für Ernährung (DGE), Österreichische Gesellschaft für Ernährung (ÖGE), Schweizerische Vereinigung für Ernährung (SGE).

[15] Hartmann B, Bell S (2006). Der Bundeslebensmittelschlüssel: Aktuelle Entwicklungen, Potential und Perspektiven; Ernährungs-Umschau : Forschung & Praxis.

[16] American Heart Association (2016). Recommended dietary pattern to achieve adherence to the American Heart Association/American College of Cardiology (AHA/ACC) guidelines: a scientific statement from the American Heart Association. Circulation.

[17] Jepsen JR, Rasmussen HB (2016). The metabolic syndrome among Danish seafarers: a follow-up study. Int Marit Health..

[18] Cassels S (2006). Overweight in the Pacific: links between foreign dependence, global food trade, and obesity in the Federated States of Micronesia. Glob Health.

[19] Micha R, Khatibzadeh S, Shi P, Fahimi S, Lim S, Andrews KG (2014). Global, regional, and national consumption levels of dietary fatsand oils in 1990 and 2010: a systematic analysis including 266 country-specific nutrition surveys. BMJ.

[20] Charlton KE, Russell J, Gorman E, Hanich Q, Delisle A, Campbell B (2016). Fish, food security and health in Pacific Island countries and territories: a systematic literature review. BMC Public Health.

[21] Zyriax B-C, Boeing H, Windler E (2005). Nutrition is a powerful independant risk factor for coronary heart disease in women - the CORA study: a population-based case-control study. Eur J Clin Nutr.

[22] Hjarnoe L, Leppin A (2013). A risky occupation? (un)healthy lifestyle behaviors among Danish seafarers. Health Promot Int.

[23] Oldenburg M (2014). Risk of cardiovascular diseases in seafarers. Int Marit Health..

[24] Kuo LE, Kitlinska JB, Tilan JU, Li L, Baker SB, Johnson MD (2007). Neuropeptide Y acts directly in the periphery on fat tissue and mediates stress-induced obesity and metabolic syndrome. Nat Med.

[25] Marmot M (2005). Social determinants of health inequalities. Lancet.

[26] Møller Pedersen SF, Jepsen JR (2013). The metabolic syndrome among Danish seafarers. Int Marit Health..

[27] Wang D, Hawley NL, Thompson AA, Lameko V, Reupena MS, McGarvey ST, et al. Dietary patterns are associated with metabolic outcomes among adult Samoans in a cross-sectional study. J Nutr. 2017; 10.3945/jn.116.243733. 10.3945/jn.116.243733 PMC5368585 28202634

[28] Seakins JM, Elliott RB, Quested CM, Matatumua A (1987). Lactose malabsorption in Polynesian and white children in the south west Pacific studied by breath hydrogen technique. Br Med J (Clin Res Ed).

[29] Saarni H, Laine M, Niemi L (2001). Health promotion in the Finnish shipping industry. Int Marit Health.

